# Usefulness of procalcitonin (PCT), C-reactive protein (CRP), and white blood cell (WBC) levels in the differential diagnosis of acute bacterial, viral, and mycoplasmal respiratory tract infections in children

**DOI:** 10.1186/s12890-021-01756-4

**Published:** 2021-11-26

**Authors:** Yang Li, Lanfang Min, Xin Zhang

**Affiliations:** Department of Clinical Laboratory, Children’s Hospital of Soochow University, Suzhou, 215025 China

**Keywords:** Children, ARTI, Inflammatory indicators, Bacterial infection, Differential diagnosis, PCT

## Abstract

**Background:**

There is a lack of studies comparing PCT, CRP and WBC levels in the differential diagnosis of acute bacterial, viral, and mycoplasmal respiratory tract infections. It is necessary to explore the correlation between above markers and different types of ARTI.

**Methods:**

108 children with confirmed bacterial infection were regarded as group A, 116 children with virus infection were regarded as group B, and 122 children with mycoplasmal infection were regarded as group C. The levels of PCT, CRP and WBC of the three groups were detected and compared.

**Results:**

The levels of PCT, CRP and WBC in group A were significantly higher than those in groups B and C (*p* < 0.05). The positive rate of combined detection of PCT, CRP and WBC was significant higher than that of single detection. There was no significant difference in PCT, CRP and WBC levels between the group of G^+^ bacterial infection and G^−^ bacterial infection (*p* > 0.05). ROC curve results showed that the AUC of PCT, CRP and WBC for the diagnosis of bacterial respiratory infections were 0.65, 0.55, and 0.58, respectively.

**Conclusions:**

PCT, CRP and WBC can be combined as effective indicators for the identification of acute bacterial or no-bacterial infections in children. The levels of PCT and CRP have higher differential diagnostic value than that of WBC in infection, and the combined examination of the three is more valuable in clinic.

## Background

Pneumonia caused by the acute respiratory tract infections (ARTI) in children has acute onset, rapid development and high mortality. It is the disease with the highest hospitalization rate and mortality of children under 5 years old [1]. Studies have found that ARTI is related to bacteria, virus and mycoplasma, among which viral infection is the most common, accounting for about 90% [2]. However, the early symptoms of children with different types of infection are fever, cough, nasal congestion, runny nose and so on. The specificity of these clinical manifestations is low, which is not conducive to clinical differential diagnosis and treatment. Failure to timely and accurately determine the type of infection leads to increased unnecessary antibiotic exposure and possible antibiotic resistance [3]. Therefore, it is very important to find an accurate and valid method for the differential diagnosis of ARTI. White blood cell (WBC) has been widely used in the diagnosis of infectious diseases. In recent years, inflammatory indicators such as Procalcitonin (PCT) and C-reactive protein (CRP) have received more and more attention in the field of differential diagnosis of ARTI [4, 5]. PCT and CRP have a good correlation with disease activity, and can be a good indication of the type of infection [6–8]. Although there are some studies on above indicators as infection markers, the comparative analysis of PCT, CRP and WBC among patients with bacterial, viral and mycoplasmal ARTI is rare.

Herein, the levels of PCT, CRP and WBC of children with different types of ARTI in Children's Hospital of Soochow University from February 2020 to March 2021 were detected. This study is to analyze the correlation between the type of ARTI and the level of PCT, CRP and WBC, so as to provide reference for the auxiliary differential diagnosis and accurate medication guidance of children who have ARTI.

## Methods

### General information

This retrospective study investigated 358 children with ARTI who were hospitalized in the Children's Hospital of Soochow University from March 2020 to February 2021. All children with ARTI have typical clinical symptoms, including fever, cough, shortness or difficulty of breath and other symptoms. The diagnostic criteria are mainly based on the handbook: Integrated Management of Childhood Illness by World Health Organization [9]. 108 children with bacteria detected by sputum culture were treated as group A, 61 males and 47 females, age (2.84 ± 3.30) years old. 116 children with common respiratory virus detected by serological test were treated as group B, 54 males and 62 females, age (4.08 ± 3.28) years old. 122 children with *Mycoplasma pneumoniae* detected by serological test were treated as group C, 54 males and 68 females, age (4.04 ± 3.14) years old. There was no significant difference between the gender of three groups (*p* > 0.05). Sputum and blood samples were collected before antibiotic treatment and transferred to the laboratory as soon as possible.

### Specimen testing

The sputum was tranferred in plates and incubated at 37℃ for 18–24 h (5% CO_2_), Then the colonies were identified by mass spectrometer. Indirect immunofluorescence method was used to detect IgM antibodies of common respiratory virus according to the instructions of the kit. The virus includes *adenovirus, respiratory syncytial virus, influenza virus A, influenza virus B and human parainfluenza virus*. Direct chemiluminescence method was applied to detect IgM antibody of *Mycoplasma pneumoniae*. 6 mL elbow venous blood was collected and injected into the coagulant tube and the anticoagulant tube containing ethylene diamine tetraacetic acid. The serum PCT level was detected by electrochemiluminescence immunometric assay, and the level not exceeding 0.5 ng/mL was considered normal [10]. The CRP and WBC level was measured by immunoturbidimetry assay and flow cytometry, respectively. According to the instructions of manufacturer, the value of CRP more than 8 mg/L was determined positive, and the value of WBC above 10 × 10^9^/L was considered positive.

### Instruments and reagents

Carbon dioxide incubator was purchased from Panasonic (MCO-18AC, Japan). Mass spectrometer was purchased from Bruker (Microflex LT/SH, Germany). Fluorescence microscope was provided from Leica (DM 2000, Germany). Chemiluminescence immunoassay analyzer was purchased from YHLO (iFlash 3000, China). Electrochemiluminescence automatic immunoanalyzer and matching reagent was purchased from Roche (Cobas E411, Germany). Automatic blood cell analyzer matching reagent was purchased from Mindray (BC-5310CRP, Shenzhen). The detection kits of virus and mycoplasma pneumoniae IgM antibody are the product of VIRCELL and YHLO, respectively. All kinds of culture plates were purchased from Antu (Zhenzhou, China).

### Statistical analysis

The statistical software of SPSS 20.0 was used for data analysis. The measurement data were expressed by mean and standard deviation (${\overline{\text{x}}} \pm {\text{s}}$), the comparison among groups was tested by u-mann whitney test. The count data were expressed by rate or composition ratio (%), and the comparison among groups was tested by χ^2^*-*test. Draw Receiver operating characteristic curve (ROC) and calculate the value of area under the curve (AUC). The difference was statistically significant with *p* < 0.05.

## Results

### Comparison of PCT, CRP and WBC levels among the three groups

The levels of PCT, CRP and WBC in group A were significantly higher than those in group B and group C (*p* < 0.05). CRP level of group C is slightly higher than group B. There was no significant difference in the levels of PCT, CRP and WBC between group B and group C (*p* > 0.05). See Table 1 for details.

**Table 1:** Comparison of PCT, CRP and WBC levels

Group	n	PCT		CRP		WBC	
		M	Q	M	Q	M	Q
A	108	0.54	1.56	8.21	29.34	8.97	5.27
B	116	0.21	0.44	4.94	10.54	8.48	4.27
C	122	0.15	0.39	3.73	18.35	8.28	4.57
*Z*_*A–B*_	–	− 3.499		− 1.345		− 1.791	
*p*_*A–B*_	–	< 0.001		0.178		0.073	
*Z*_*A–C*_	–	− 4.634		− 1.752		− 2.891	
*p*_*A–C*_	–	< 0.001		0.080		0.004	
*Z*_*B–C*_	–	− 1.090		− 0.148		− 0.913	
*p*_*B–C*_	–	0.276		0.882		0.361	

*Z*_*A–B*_ represents the *Z*-value of *u*-mann whitney test between group A and group B. *p*_*A-B*_ represents the p-value of u-mann whitney test between group A and group B. The same goes for *Z*_*A-C*_, *p*_*A-C*_, *Z*_*B-C*_, *p*_*B-C*._ *M* represents the median. *Q* represents the interquartile range

### Comparison of positive rates of PCT, CRP and WBC among the three groups

The positive rates of PCT and CRP in group A were significantly higher than those in group B and group C. The differences of PCT and CRP levels among the three groups were statistically significant (*p* < 0.05). There was no significant difference in the positive rate of WBC among the three groups (*p* > 0.05). The combined diagnosis of three indicators can significantly improve the positive rate of diagnosis, as shown in Table 2.

**Table 2:** Comparison of positive rates of PCT, CRP and WBC [n (%)]

Group	n	PCT	CRP	WBC	Combined diagnosis
A	108	57 (52.78)	55 (50.93)	42 (38.89)	94 (87.04)
B	116	25 (21.55)	43 (37.07)	36 (31.03)	78 (67.24)
C	122	29 (23.77)	42 (34.43)	34 (27.87)	78 (63.93)
*χ*^*2*^_*A*_–_*B*_–_*C*_	–	31.00	12.07	3.32	17.44
*p*_*A*_–_*B*_–_*C*_	–	< 0.001	0.002	0.190	< 0.001
*χ*^*2*^_*A*_–_*B*_	–	22.50	4.364	1.52	12.26
*p*_*A*_–_*B*_	–	< 0.001	0.037	0.218	< 0.001
*χ*^*2*^_*A*_–_*C*_	–	20.59	5.07	3.14	16.21
*p*_*A*_–_*C*_	–	< 0.001	0.024	0.076	< 0.001
*χ*^*2*^_*B*_–_*C*_	–	0.17	19.35	0.29	0.29
*p*_*B*_–_*C*_	–	0.683	< 0.001	0.592	0.592

*χ*^2^_*A*_–_*B*_–_*C*_ represents the *χ*^2^-value of chi square test between group A, group B and group C. *p*_*A*_–_*B*_–_*C*_ represents the *p*-value of chi square test between group A, group B and group C. The same goes for *χ*^2^_*A*_–_*B*_, *p*_*A*_–_*B*;_ *χ*^2^_*A*_–_*C*_, *p*_*A*_–_*C*_; *χ*^2^_*B*_–_*C*_, *p*_*B*_–_*C*_

### ROC curve analysis of PCT, CRP and WBC in the diagnosis of bacterial ARTI

**Fig. 1:**
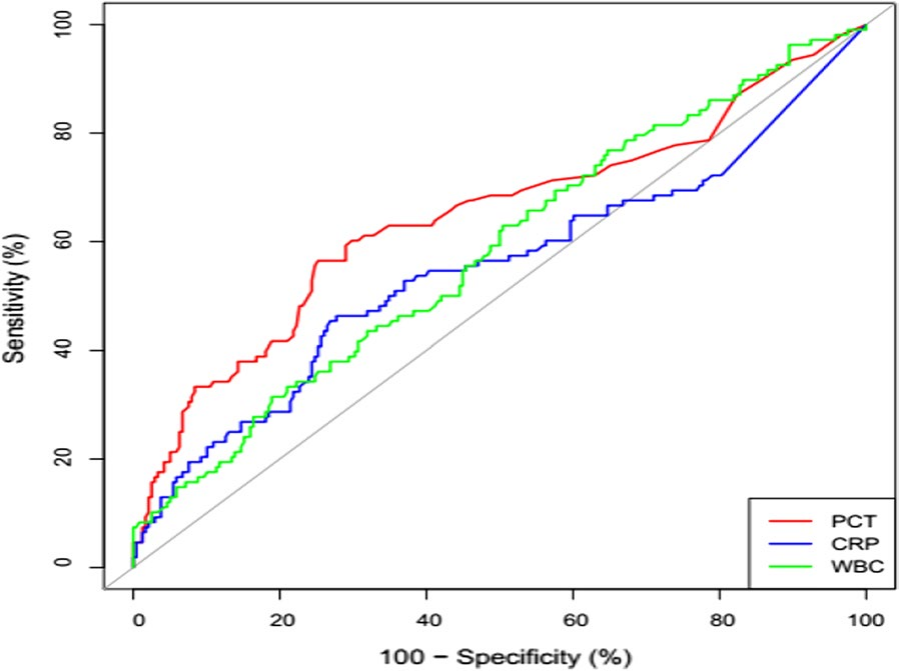
ROC curve of PCT, CRP and WBC in diagnosis of bacterial ARTI

ROC curve analysis showed that the areas under the curve of PCT, CRP and WBC were 0.65, 0.55 and 0.58 respectively. See Table 3 and Fig. 1 for details.

**Table 3:** The efficacy of PCT, CRP and WBC in the diagnosis of bacterial ARTI

Index	AUC (95% confidence interval)	Cut-off	Specificity (%)	Sensitivity (%)	Positive predictive value (%)	Negative predictive value (%)
PCT	0.65 (0.59–0.71)	0.49	74.80	56.5	50.4	79.1
CRP	0.55 (0.48–0.62)	10.63	72.3	46.3	43.1	74.8
WBC	0.58 (0.52–0.65)	11.40	81.1	31.5	43.0	72.3

### Distribution of bacterial respiratory tract infection strains

The types of bacteria detected in 108 children of bacterial ARTI were as follow: gram-positive (G^+^) bacteria were mainly *S. pneumoniae* and *S. aureu*s, gram-negative (G^−^) bacteria were mainly *E. coli* and *H. influenzae*. As shown in Table 4.

**Table 4:** Distribution of bacterial respiratory tract infection strains

Type	n	Percentage (%)
**G**^**+**^** bacteria**	**60**	**55.56**
*S. pneumoniae*	33	30.56
*S. aureus*	26	24.07
*S. agalactiae*	1	0.93
**G**^**−**^** bacteria**	**48**	**44.44**
*E. coli*	12	11.11
*H. influenzae*	10	9.26
*M. catarrhalis*	7	6.48
*A. baumannii*	6	5.56
*P. aeruginosa*	5	4.63
*K. pneumoniae*	4	3.70
*Burkholderia*	2	1.85
*E. aerogenes*	1	0.93
*S. maltophilia*	1	0.93

Bold indicates G^+^ bacteria represents Gram-positive bacteria, G^−^ bacteria represents Gram-negative bacteria

### Clinical diagnosis of patients infected with common bacterial infection

Statistics show that there are some common and rare bacteria. Common bacteria usually include *S. pneumoniae, S. aureus, E. coli, H. influenzae* and so on*.* Rare bacteria are *S. agalactia*e, *Burkholderia, E. aerogene and S. maltophilia* in this study. Table 5 shows the corresponding clinical diagnostic information of patients with bacterial infection.

**Table 5:** Diagnosis of patients infected with common and rare bacteria

Clinical diagnoses	Common bacteria								Rare bacteria				Total
	*S. pneumoniae*	*S. aureus*	*E. coli*	*H. influenzae*	*M. catarrhalis*	*A. baumannii*	*P. aeruginosa*	*K. pneumoniae*	*Burkholderia*	*E. aerogenes*	*S. maltophilia*	*S. agalactiae*	
Pneumonia	23	17	7	7	6	3	2	1	0	0	1	0	67
Upper respiratory tract infection	3	1	0	2	0	0	0	0	0	0	0	1	7
Other infections	5	2	0	0	0	0	0	0	0	0	0	0	7
Fever	2	4	0	0	1	0	0	0	0	0	0	0	7
Heart disease	0	0	1	0	0	1	0	1	0	1	0	0	4
Asphyxia	0	0	0	0	0	0	1	1	0	0	0	0	2
Jaundice	0	0	2	0	0	0	0	0	0	0	0	0	2
Intestinal obstruction	0	0	0	0	0	0	1	0	1	0	0	0	2
Epilepsy	0	1	1	0	0	0	0	0	0	0	0	0	2
Tumor	0	0	1	0	0	0	0	0	0	0	0	0	2
Kawasaki disease	0	0	0	0	0	0	0	0	0	0	0	0	0
Other diagnosis	0	1	0	1	0	2	1	1	1	0	0	0	6
Total	33	26	12	10	7	6	5	4	2	1	1	1	108

### Comparison of PCT, CRP and WBC levels and diagnostic positive rate between G^+^ and G^−^ bacterial infection group

There were no significant differences in PCT, CRP, WBC levels and diagnostic positive rates between G^+^ and G^−^ bacterial infection groups (*p* > 0.05), as shown in Table 6.

**Table 6:** Comparison of PCT, CRP and WBC levels and diagnostic positive rate between G^+^ and G^−^ bacterial infection group

Group	n	PCT		CRP		WBC		Combined diagnosis
		M	n (%)	M	n (%)	M	n (%)	
G^+^ bacteria	60	0.57	34 (56.67)	8.37	31 (51.67)	8.97	21 (35.00)	52 (86.67)
G^−^ bacteria	48	0.50	23 (47.92)	7.87	24 (50.00)	9.30	21 (43.75)	40 (83.33)
*Z*	–	− 0.124	–	0.797	–	0.269	–	–
*p*	–	0.902	–	0.426	–	0.788	–	–
*χ*^*2*^		–	0.82	–	0.03	–	0.86	0.24
*p*		–	0.37	–	0.86	–	0.35	0.63

*M* represents the median

### Clinical information

At the same time, the clinical information of 346 patients was also analyzed according to different department, age, gender and clinical diagnoses. The results are shown in Table 7.

**Table 7:** Clinical information

	Classification	A (n = 108)	B (n = 116)	C (n = 122)
Department	Respiratory	36	36	55
	Intensive Care Unit	25	1	1
	Infectious Diseases	18	30	17
	Neonatology	22	0	0
	Renal Immunology	2	8	8
	Emergency Medicine	2	6	1
	Neurology	1	0	4
	Urology	1	0	0
	Hematology	1	0	3
	Internal Medicine General Ward	0	13	8
	Division of Rheumatology	0	10	9
	Cardiology	0	12	12
	Endocrinology	0	0	3
	Gastroenterology	0	0	1
Age	0 ~ < 1Y	40	13	14
	1 ~ < 3Y	23	29	35
	3 ~ < 14Y	43	73	72
	14 ~ < 18Y	2	1	1
Gender	Male	61	54	54
	Female	47	62	68
Clinical diagnoses	Pneumonia	67	34	44
	Upper respiratory tract infection	7	41	32
	Other infections	7	18	13
	Fever	7	14	13
	Heart disease	4	0	1
	Asphyxia	2	0	1
	Jaundice	2	0	0
	Intestinal obstruction	2	0	0
	Epilepsy	2	0	1
	Tumor	2	0	0
	Kawasaki disease	0	0	3
	Other diagnosis	6	9	14

## Discussions

ARTI is the most common infectious disease in children, and has a significant impact on children's health [11]. Diagnosis of the type of bacterial, viral or mycoplasmal infection mainly need the support of bacterial culture, nucleic acid test or antibody detection. The culture process is time-consuming and tedious, and the results can not be fed back to the clinic in time. Nucleic acid test may cause missed detection due to sample collection problems. Antibody detection is affected by factors such as infection time, antibody concentration and insufficient sensitivity of the detection method. For different types of infections, clinical medications are also different. Experiential medication is a common clinical treatment, and it is also one of the causes of antimicrobial drug abuse and bacterial resistance [12]. It is an urgent problem to quickly distinguish the types of infection and guide clinical accurate drug use.

WBC count in blood routine is a common method in clinical diagnosis of infection [13]. It is easy to operate and the result is fast. However, it is easy to be interfered by external factors, which affects the choice of clinical medication. Both PCT and CRP are acute phase reaction proteins, they can change according to the level of inflammatory factors. Especially PCT, it can be activated by microbial toxins, interleukin-1, inteleukin-6, and tumor necrosis factor-α [14]. Conversely, PCT is inhibited by the interferon-γ factor released by the virus [15]. The level of PCT in serum increases significantly, when the body is infected with bacteria. The more serious of the bacterial infection, the higher the PCT level [16]. CRP is a non-specific acute phase reaction protein synthesized by hepatocytes. As an inflammatory marker, it is easy to detect and has high accuracy. It is widely used in the diagnosis and prognosis of patients with acute bacterial infection [17, 18]. Therefore, in view of the time-consuming bacterial culture, the advantages of rapid identification of PCT and CRP can be used to quickly identify the types of ARTI before the culture results are obtained, so as to provide meaningful reference data for clinical practice.

The results of this study show that the levels of PCT and CRP in group A were significantly higher than those in group B and group C (*p* < 0.05). It’s clear that bacterial infection can lead to elevated levels of above indicators. A study involving seven countries showed that the increase of CRP was positively correlated with bacterial pneumonia and negatively correlated with pneumonia infected by a virus [19]. And a study on PCT showed it increased significantly during bacterial infection [20]. This may be related to the failure of PCT to break down into calcitonin under the action of cytokines during bacterial infection, resulting in the increase of PCT level in blood [21]. Results of this study are consistent with the relevant reports [22, 23]. When PCT, CRP or WBC remarkably increased, the possibility of bacterial infection increased significantly. It can guide the clinical early use of broad-spectrum cephalosporins or aminoglycoside antibiotics for treatment, and can achieve ideal therapeutic effect. Then the positive rates of PCT, CRP and WBC among the three groups were compared. The positive rates of PCT and CRP in group A were significantly higher than those in group B and group C (*p* < 0.05). The result is consistent with the reported by Feng et al. [5], when patient infected with bacteria, the level of PCT and CRP in blood would increase significantly. There was no significant difference in the positive rate of WBC among the three groups (*p* > 0.05). More importantly, the study found that the combined diagnosis of three indicators can significantly improve the positive rate of diagnosis, and the result is consistent with most reported studies [24, 25]. This suggests that clinical multi-index joint detection is very important for the diagnosis of disease. To further analyze the diagnostic efficacy of PCT, CRP and WBC using ROC curve for bacterial ARTI. The result showed that the AUC of PCT, CRP and WBC were 0.65, 0.55 and 0.58, respectively. And the sensitivity of PCT, CRP and WBC were 56.5%, 46.3% and 31.5%, respectively. Overall, it prompted that the diagnostic value of PCT and CRP was better than that of WBC in bacterial infection, while WBC was not very useful. It was consistent with the relevant studies [26, 27]. The sensitivity of PCT in this study was 56.6%, which was lower than that of a study evaluating the accuracy of PCT in identifying viral and bacterial pathogens, with a sensitivity of 80.9% [28].

Because the preferred antibiotics for different bacterial infections are not same, single broad-spectrum antibiotic treatment is difficult to continuously control the development of the disease. Therefore it is necessary to further explore the types of bacterial infections. Among 108 children with bacterial ARTI, there were 60 cases of G^+^ bacteria and 48 cases of G^−^ bacteria. As shown in Table 4, G^+^ bacteria were mainly *S. pneumoniae* and *S. aureus*, G^−^ bacteria were mainly *E. coli* and *H. influenzae* and so on. As we all know, these bacteria are common pathogens of ARTI, and the result is consistent with the reported of Zong et al. [29]. Children are very vulnerable to infection due to their poor development and low immunity. Next, the levels and diagnostic positive rate of PCT, CRP and WBC between G^+^ and G^−^ bacterial infection group were depth comparative analyzed. As shown in Table 6, three indicators levels of G^−^ bacteria group are slightly higher than that of G^+^ bacteria group. However, there were no significant differences in PCT, CRP, WBC levels and diagnostic positive rates between G^+^ and G^−^ bacterial infection groups (*p* > 0.05). This result is a little different from that of Tang et al. [6], which may be due to insufficient cases included in this study. In Table 7, the respiratory and infectious diseases department had the highest proportion of patients with ARTI. Children aged 3 ~  < 14 Y were at greater risk of infection to pathogen, which was consistent with the survey report of Ma et al. [30]. The clinical symptoms corresponding to ARTI, such as pneumonia, upper respiratory tract infection and fever, occupy a relatively high proportion.

## Conclusions

According to the above results, PCT, CRP and WBC are helpful to distinguish acute bacterial or no-bacterial infections in children to a certain extent, and their levels can prompt clinicians about infection and try to avoid the abuse of antibiotics. The differential diagnosis effect of PCT and CRP is better than that of WBC. The combined diagnosis of PCT, CRP and WBC can significantly improve the positive rate of diagnosis. However, there are some limitations in this study. Such as, the number of samples involved in this study is not enough, and there is a lack of control group to strengthen statistical robustness. So we will pay attention to in the follow-up study. In general, this study provides a new idea for the diagnosis and differential diagnosis of ARTI.

## Data Availability

The data are available from the corresponding author on reasonable request.

## Abbreviations

PCT: Procalcitonin
CRP: C-reactive protein
WBC: White blood cell
ARTI: Acute respiratory tract infections
ROC: Receiver operating characteristic curve
AUC: Area under the curve
G^+^: Gram-positive
G^−^: Gram-negative
